# Self-Assessment of Health Status and Willingness to Be Vaccinated in Adolescents from the Niigata Prefecture and the Khabarovsk Region during COVID-19

**DOI:** 10.3390/healthcare10020184

**Published:** 2022-01-18

**Authors:** Hiromi Inaba, Marina F. Rziankina, Fumi Hoshino, Kousuke Takano, Kseniia E. Potapova, Konstantin V. Zhmerenetsky, Kazuo Ishigami

**Affiliations:** 1 Center for Nutrition Sciences, Niigata University of Health and Welfare, Niigata 951-8102, Japan; 2 Department of Health and Nutrition, Faculty of Health Science, Niigata University of Health and Welfare, Niigata 951-8102, Japan; fumi-h@nuhw.ac.jp; 3 Department of Polyclinic Pediatrics with a Course of Children’s Infectious Diseases, Far Eastern State Medical University, 680000 Khabarovsk, Russia; rzyankina@mail.ru (M.F.R.); pediatr27@yandex.ru (K.E.P.); zhmerenetsky@list.ru (K.V.Z.); 4 Department of Health Information, Niigata University of Health and Welfare, Niigata 951-8102, Japan; kousuke-takano@nuhw.ac.jp (K.T.); ishigami@nuhw.ac.jp (K.I.)

**Keywords:** COVID-19, adolescent, mental health, new lifestyle, vaccinate

## Abstract

This study examined the self-assessment of Niigata’s and Khabarovsk’s adolescents’ health status and their willingness to be vaccinated during the COVID-19 pandemic. A self-reported questionnaire was administered to 735 boys and girls (aged 15 years) from Niigata, Japan (*n* = 387), and Khabarovsk, Russia (*n* = 394), between May and July 2021. Specifically, this questionnaire focused on COVID-19, including a self-assessment of health status, adaptation to a new lifestyle, and impressions about the COVID-19 vaccination. The self-assessment was based on a 4-point scale: “Got very bad”; “Got a little bit bad”; “Did not change”; “Got better/I don’t know”. Additionally, binomial logistic regression was conducted to determine the association between the self-assessment of health status and the factors exacerbating their responses. Based on the findings, 25.7 and 29.9% of Niigata and Khabarovsk’s adolescents, respectively, selected “Got very bad” and “Got a little bit bad” for their self-assessments, while the binomial logistic regression showed that the difficulty of adapting to a new lifestyle was a factor worsening the boys’ subjective health. However, the items could not explain the deterioration of their subjective health in girls. Moreover, 76.9% of Niigata’s adolescents were positive about the COVID-19 vaccination, compared to 35.5% of the adolescents in Khabarovsk.

## 1. Introduction

On 11 March 2020, the World Health Organization (WHO) declared the COVID-19 outbreak a global pandemic [1]. Since then, there have been 253 million confirmed cases, including 5.1 million deaths [2]. The COVID-19 pandemic has also greatly changed people’s daily lives, forcing countries to take actions such as lockdowns, quarantines, and social distancing measures [3].

In general, the total number of patients with COVID-19 is reported daily by each institution [2,4,5]. Meanwhile, infection among children and adolescents is generally less severe and causes fewer deaths than in adults [6,7]. Consequently, less attention is being paid to pediatric patients due to the milder course of the virus. Previous research has confirmed that the risk of developing severe and critical conditions in children is much lower than that in adults [8]. However, there is a concern that the number of tests will be reduced; consequently, the number of infected children and adolescents will be lower than the actual number. According to publicly available data from Khabarovsk and other countries’ sources, the clinical picture of COVID-19 infection in children is mostly characterized by lack of pathology, lack of specificity, instability, and lack of manifestations. In fact, the most common clinical signs of the virus among children living in Khabarovsk were fever (47.3%) and nonproductive cough (31.2%), followed by impaired nasal breathing in the form of nasal congestion and coryza (20.2%), and anosmia (10.4%) [9].

As for other aspects, the pandemic has changed not only adults’ lives but also the daily lives of children. For example, schools were shut down, and education was provided through remote learning. Even when children were able to attend school, most events, such as school trips, athletic meets, and cultural festivals, were canceled. There have also been numerous reports on the deterioration of children’s mental health [10,11], with disruptions to their sleep rhythms, eating habits, and physical activities [12]. For example, Pieh et al. reported that 18.6% of young adults were suspected of having insomnia during lockdown [13].

In Japan, suicides among high school adolescents have significantly increased, reaching 46 in August 2021, which is 2.1 times that of August 2020 [14]. In particular, the suicide rate among high school girls has risen from 4 to 23 [14]. Magson et al. reported that high school students are more concerned about maintaining self-restraint than being infected with COVID-19 itself, and such concerns can increase anxiety and depression [15].

In regard to ending the COVID-19 pandemic, vaccinations are essential. Although vaccination of the elderly and healthcare workers at high risk of infection has been a priority in many countries, the situation regarding children has varied from country to country. To ensure that children are vaccinated, we believe it is necessary to survey their willingness to be vaccinated. Unfortunately, it is estimated that it will take longer for the COVID-19 infection to resolve. Thus, it is important to clarify the factors related to the health maintenance of children/adolescents during this pandemic.

For this purpose, we considered two cities with similar situations: Niigata City and the Khabarovsk Region. With a population of 800,000, Niigata City—which is the capital of Niigata Prefecture, Japan—is an appropriate subject region for our research. Khabarovsk City, Russia, was chosen as the subject because of the similarities: the population of 600,000, and because it is the capital of the Khabarovsk region. What both cities have in common is that they are the core cities of the region. As core cities, they also have similar medical systems, such as public cancer centers and hospices. As Niigata City and Khabarovsk City are sister cities that have continued for more than 50 years, comparative studies of adolescence [16] and old age [17] in both cities have been conducted for some time.

Therefore, in this study, we examined the self-evaluation of the health status of adult adolescents in Niigata City and Khabarovsk City and the intention of vaccination during the COVID-19 pandemic.

## 2. Materials and Methods

### 2.1. Participants

A cross-sectional survey was administered to a total of 735 boys and girls (aged 15 years) from Niigata, Japan (*n* = 387), and Khabarovsk, Russia (*n* = 394). The population of 15-year-olds in Niigata City was 6724 (as of 31 July 2021), and the 387 people surveyed represented 5.76% of the total population of 15-year-olds. In contrast, the population of 15-year-olds in Khabarovsk City was 10,534 (as of 3 May 2021), and the 394 people surveyed represented 3.74% of the total population of 15-year-olds. The subject senior high school, hereby H High School, is one of the largest private high schools in Niigata, with 440 students per grade (approximately 40 students per class). This high school was selected due to its variety of students, including students who proceed to attend national universities and exclusive private universities, students who proceed to attend mid-career private universities, and students who wish to find a job. Furthermore, as it is a private high school, the majority of the families are at a certain economic level. In Khabarovsk, 394 15-year-old students from six secondary schools of the city of Khabarovsk (Russian Federation) were enrolled. In each school, students from two 8th and 9th grades were surveyed (an average of 60 people from each). In Russia, specialized education commences in grade 10; therefore, all respondents studied according to the general education program. In total, we have 2063 students from one private high school in Niigata Prefecture and six schools in Khabarovsk City (Niigata; 1343, Khabarovsk; 720). For the target population of students, a sample of at least 360 students is required to explore the variables selected for this study, assuming a response rate of 50%, a 95% confidence level, and a 5% margin of error, as discussed in previous studies [18].

There are two reasons why we selected 15-year-olds. First, we have studied the perceptions of unhealthy body shape, dieting habits, and eating habits among 15-year-old adolescents. This is because 15-year-olds are included in the target age group of the Health Behavior of School Children (HBSC) survey (11, 13, and 15 are considered the target ages). This survey is conducted every four years by 50 countries and regions across Europe and North America in collaboration with the WHO, and we wanted to compare this study internationally in the future. Second, research into adolescents’ subjective health and vaccination study is reported less than that for adults and older people.

Permission was obtained from the principals of the schools in both cities and the students were informed about the details of the study. Subsequently, a briefing document was distributed to the students, after which they were asked to take the questionnaire home and only complete it when both themselves and their parents agreed to participate.

### 2.2. Survey Period

This study was conducted during a period when the momentum of infection was intense in both countries. Specifically, the survey in the Khabarovsk region was conducted in May 2021 (when the number of newly infected people was approximately 10,000 per day), and that in the Niigata Prefecture in July 2021 (approximately 1000 people per day).

### 2.3. Questionnaire and Anthropometry

The questionnaire used in this study was first prepared in Khabarovsk. Five coworkers—one professor, three associate professors, and a teaching assistant (an assistant professor)—from the Department of Polyclinic Pediatrics with a Course of Children’s Infectious Diseases (FSBEI HE “Far Eastern State Medical University” of the Ministry of Health of the Russian Federation tested the questionnaire and incorrect questions were excluded from the questionnaire. These academics rejected incorrect questions from the questionnaire and selected the optimal questions for the survey. Subsequently, the questionnaire was translated into Japanese by a Russian-based interpreter who specializes in medical interpretation. Subsequently, the translated questionnaire was further checked by a translator living in Japan to remove inappropriate expressions. In addition, in Niigata, five university students were asked to check whether there were any questions that were difficult for high school students to answer (these data were excluded from the paper).

The questionnaire in this study was related to COVID-19, gender, height, and weight, after which their body mass index (BMI) was calculated. In addition, a 5-point-scale (About every day; More than once a week; About every week; About every month, and Rarely or Never) survey was conducted on the health status of eight items selected from the HBSC questionnaire when reviewing the previous six months. The questions involved headache, stomachache, back pain, depression, frustration, nervousness, sleep difficulties, and dizziness. The specific content of the questionnaire included: the self-assessment of health status (Got very bad; Got a little bit bad; Did not change; Got better/or I don’t know), adaptation to a new lifestyle (I didn’t feel much difference so I didn’t have any problem; I adapted comfortably so I didn’t have any problem; It was difficult to adapt but I adapted a little; I couldn’t adapt), and the fear of infection (Yes, very scary; somewhat scary, I’m scared but I’m ready to get infected; not too scary; I think it’s just a cold). In addition, the “change in eating habits,” “exercise habits,” and the impressions of the COVID-19 vaccination were surveyed.

### 2.4. Statistical Analysis

The data were entered into the R statistics program (Version 4.0.2.) [19]. The analysis was conducted on 721 participants (327 in Niigata and 394 in Khabarovsk) who responded to the self-assessment of health status. For country differences, we used the chi-square test for the nonparametric variables. As stated earlier, height, weight, and BMI were *t*-tested between the two countries. Additionally, binomial logistic regression (by gender) was conducted to determine the association between the participants’ self-assessment of health status and the factors that exacerbated their responses. The objective variables included self-assessment of health status, while the explanatory variables included adaptation to a new lifestyle, regular lifestyle, appropriate exercise, and impressions about the COVID-19 vaccination. The explanatory variables were selected by the forward and backward stepwise method, while we calculated the odds ratio (OR), with a 95% confidence interval for the factors that exacerbated the self-assessment of health status. Multicollinearity was assessed using the variance inflation factor (VIF); in this case, a VIF exceeding 10 was regarded as serious multicollinearity, while values greater than 4.0 might be a cause for concern. The level of significance was set at 0.05 for each analysis.

### 2.5. Ethical Considerations

This study complies with the Declaration of Helsinki and was approved by the institutional review boards of both the Niigata University of Health and Welfare (Approval No. 18594-210412) and the Far East Medical University (Approval No. 0128042021). As stated earlier, a briefing document was distributed to the students, after which they were asked to take the questionnaire home and only complete it when both they and their parents agreed to participate. The survey instructions included the purpose of the survey, the content and processing of the information obtained, and statements that their participation was voluntary and that there was no disadvantage in refusing to participate.

## 3. Results

The physical characteristics of the Niigata and Khabarovsk’s adolescents are shown in Table 1. Based on the findings, both the Niigata boys and girls were taller and heavier than their Khabarovsk counterparts, but there was no significant difference in BMI.

Table 2 shows the health condition at the time of the survey. The percentage of students who were ill was 25.4% in Niigata and 30.2% in Khabarovsk.

**Figure 1:**
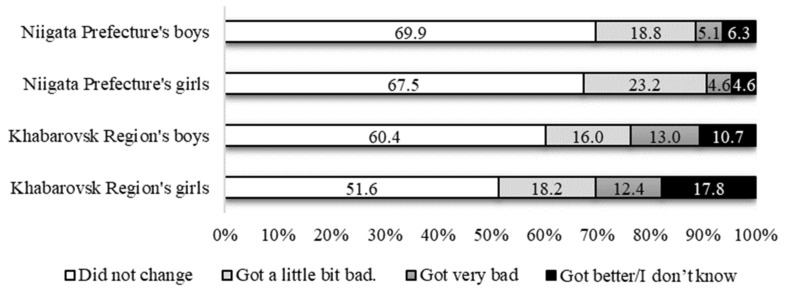
“Has the pandemic changed your self-assessment of health status?” (*n* = 721).

Table 3 shows the self-reported health status of the subjects for the previous six months. Both boys and girls in Khabarovsk had a significantly higher percentage of nervousness than those in Niigata. In addition, Khabarovsk girls had significant difficulty falling asleep (*p* < 0.001). The results of the self-assessment of health status are shown in Figure 1. In this case, the Khabarovsk girls had the highest percentage of worsening (30.7%), followed by the Khabarovsk boys (29.0%), the Niigata girls (27.8%), and the Niigata boys (23.9%). Overall, 39.1% of the Niigata girls stated that although they were eventually successful, they had difficulty adapting to the new lifestyle, compared to only 21.3% of the Khabarovsk boys and girls.

**Figure 2:**
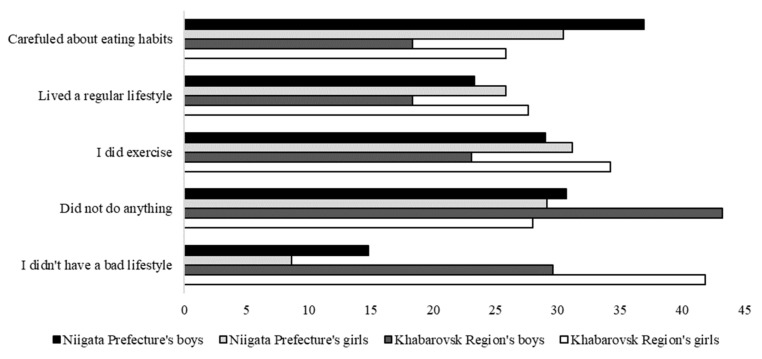
“What are you doing/not doing to improve your health?”.

As for other aspects, 8.9% of the Khabarovsk boys had difficulty tolerating self-restraint (see Table 4), while a high percentage of the Niigata students were afraid of being infected with COVID-19; that is, 64.2% boys and 78.8% girls (see Table 5). Conversely, only 14.2 and 16.0% of the Khabarovsk boys and girls, respectively, were afraid of infection. To maintain good health, a high percentage of the Niigata’s adolescents practiced correct eating habits, while 77 Khabarovsk girls (34.2%) were engaged in moderate exercise. However, in Khabarovsk, 73 boys (43.2%) did not do anything (see Figure 2).

The results of the binomial logistic regression showed that the factors exacerbating the self-assessment of health status differed between the boys and girls. For the Niigata boys, not having correct eating habits (*p* < 0.05) and having difficulty adapting to a new lifestyle (*p* < 0.05) significantly exacerbated their self-assessment of health status. In particular, not having correct eating habits resulted in an OR of 3.003 (see Table 6). For both the Khabarovsk and Niigata boys, difficulty adapting to a new lifestyle exacerbated their subjective health evaluation (*p* < 0.001). Preparing to be infected with COVID-19 also had a negative impact on their subjective health evaluation (*p* < 0.005) (see Table 7). Conversely, there were no factors that exacerbated the girls’ self-assessment of health status as opposed to the boys (see Table 8 and Table 9). Evidence of multicollinearity was absent because the VIF for the independent variables in all of the models in Table 6, Table 7, Table 8 and Table 9 was less than 4.0.

Finally, the question related to vaccine hesitancy included several response options and was clustered to inform delivery. The first response, which was (1) positively accept it or get vaccinated and will vaccinate when asked to vaccinate was categorized as vaccination “opt-in”; (2) those who responded do not know and not bothered were categorized as “undecided”; (3) those who were unwilling and antivaccination were categorized as “opt-out.” Overall, 718 potentially accepted the vaccine, of whom 389 (54.2%) would opt in to take the vaccination, 185 (25.8%) were undecided, and 144 (20.1%) would opt out. A higher percentage of Niigata’s adolescents reported that they would opt in to the vaccination. For example, 75.9% Niigata boys and 78.0% Niigata girls, compared to 37.3% Khabarovsk boys and 34.2% Khabarovsk girls, would opt in to take the vaccination (see Table 10).

## 4. Discussion

Niigata’s adolescents were more likely to be afraid of infection (see Figure 1), but the boys and girls who rated their self-assessment of health status as “Got very bad” and “Got a little bit bad” were less likely than those in Russia (see Table 4).

In Japan, from 2 March to 6 April 2020, temporary closures were implemented in elementary, junior high, and high schools nationwide [20]. In July 2021, when the investigation was conducted, the fourth state of emergency was being issued [20]. Accordingly, it is presumed that the fear of infection was higher than that in Khabarovsk region. In Niigata, even if one person was infected with COVID-19, then the entire school was shut down (not just the class). We speculated that this fear of infection was directly related to the subjective deterioration of the human physical condition.

In this study, the factor that worsened the self-assessment of health status of the boys in both Niigata and Khabarovsk was the difficulty of adapting to a new lifestyle. Magson et al. reported that adolescents are more concerned about the government restrictions designed to contain the spread of the virus, compared to the virus itself. Additionally, they found that these concerns were associated with increased anxiety and depressive symptoms, and decreased life satisfaction [15]. Although Khabarovsk’s adolescents had a worse self-assessment of health status, about half of the Khabarovsk boys stated that they did nothing to maintain their health (see Figure 2). In this regard, it may be possible to prevent the deterioration of the self-assessment of health status by focusing on better eating habits, as in Niigata’s adolescents.

It is important to clarify the factors that have increased the suicide rate in children/adolescents, especially among high school girls in Japan [14]. Specifically, as their parents spend more time working from home, the children are careful to not interfere with their work due to the increased stress from financial instability. Meanwhile, in addition to the long-term school closure, teachers and friends have limited time to talk, and the opportunity to discuss their concerns is lost. However, the questions in this survey were unable to clarify these factors among the girls in Niigata Prefecture.

We also believe that these were some of the reasons why Niigata’s adolescents were so positive about the vaccination. These were in addition to the strong fear of infection, a high level of trust about the vaccine, the concern that the school would be shut down, and the greater possibility that their loved ones would not get infected. According to a June 2020 survey of 19 countries with high COVID-19 rates by Lazarus et al., 54.9% answered that they were willing to vaccinate, with China having the highest rate (88.6%) and Russia having the lowest (54.9%) [21]. It has also been reported that older age groups are more positive toward the vaccination [21,22]. Because the participants in the present study were younger than those of, for example, Jeffrey et al.’s study (18 years and older), it is not surprising that the willingness to vaccinate in Khabarovsk’s adolescents was low. However, as the Russian government has made a vaccine available for general use on the basis of extremely limited data [23], we speculate that there may have been less confidence in the efficacy of the vaccine, thus decreasing the willingness to accept the vaccination.

In a related study, Cornwall pointed out that misinformation spread through multiple channels can have a considerable effect on the acceptance of a COVID-19 vaccine [24]. All of the respondents, regardless of nationality, reported that they would be less likely to accept a COVID-19 vaccine if it were mandated by employers [21]. In this regard, if children are forced to be vaccinated by their school, then there is a concern that the number of vaccinated applicants will decrease. Hence, it is necessary to consider other methods for increasing the number of vaccinated adolescents.

Montagni et al. reported that many people hesitated to vaccinate when their health literacy was low, indicating that the acceptance of vaccination differs depending on the level of health literacy [25]. Rachel et al. reported that vaccine education must be directed at people with poor health literacy and younger school-age [26]. Even in adolescence, a clarified target approach is effective in effectively increasing the vaccination rate of COVID-19.

While health literacy education at school is important for children, educating parents is essential because children spend a great amount of time at home, too. Health literacy and many of its determinants are under-researched in children and adolescents, unlike adults.

Of course, disseminating the usefulness of school education and vaccination as a national policy needs to continue, but adolescents are unnecessarily afraid of false information disseminated online. During adolescence, a huge amount of information is sent and received via smartphones and the Internet. Therefore, we believe that health literacy education is very important. It is inferred that this is true not only during the COVID-19 pandemic but also during other health education.

In Italian university students, acceptance of COVID-19 vaccination was reported to be associated with influenza vaccination rates and knowledge of COVID-19 [27]. However, in the present study, we did not confirm vaccination history other than COVID-19 or knowledge of COVID-19. By confirming the previous vaccination history and knowledge of COVID-19, we can propose an effective vaccination method for high school students in Niigata Prefecture and Khabarovsk region.

Finally, there are a few limitations in this study that should be noted. First, we only targeted one high school in Niigata and six schools in the city of Khabarovsk, located in the Far East of Russia. Second, the validity could not be examined carefully enough. Further face and content validity were needed to carry out the survey accurately. This survey did not include a question on whether the respondents had ever been infected with COVID-19. Finally, chronic diseases such as type 1 diabetes, asthma, and ulcerative colitis needed to be investigated as well. Therefore, future research should focus on a wider sample from different countries and include additional COVID-19-related questions to generalize the findings.

## 5. Conclusions

This study examined the self-assessment of Niigata and Khabarovsk adolescents’ health status and their willingness to be vaccinated during the COVID-19 pandemic. This research found that 25.7% of Niigata’s adolescents and 29.9% of Khabarovsk’s adolescents rated themselves as “bad” in their self-assessment of health status. These results indicate the importance of presenting ways in which adapting to a new lifestyle could be made easy, focusing on the importance of certain factors that reduce their subjective health assessments. In addition, it was revealed that the Niigata boys did not eat properly, while the Khabarovsk boys were negatively affected by their readiness for infection. Based on these findings, more research is required to examine the factors that exacerbate the self-assessment of health status among girls.

## Figures and Tables

**Table 1:** Physical characteristics of Niigata and Khabarovsk’s adolescents, *n* = 721.

		Boy							Girl						
		Niigata Prefecture			Khabarovsk Region			*p*-Value	Niigata Prefecture			Khabarovsk Region			*p*-Value
		*n* = 176			*n* = 169				*n* = 151			*n* = 225			
Height	cm	170.5	±	6.2	172.6	±	9.0	0.011	158.4	±	5.4	163.8	±	7.0	<0.001
Weight	kg	60.5	±	9.0	63.1	±	17.3	0.087	49.7	±	6.1	54.5	±	9.5	<0.001
BMI	kg/m^2^	20.8	±	2.8	21.0	±	5.0	0.594	19.8	±	2.1	20.3	±	3.1	0.092

BMI: body mass index.

**Table 2:** Health status at the time of the survey, *n* = 721.

Health Status	Niigata Prefecture				Khabarovsk Region			
	Boy	Girl	Total		Boy	Girl	Total	
	*n* = 176	*n* = 151	*n* = 327		*n* = 169	*n* = 225	*n* = 394	
Excellent	27	18	45	(13.8)	36	32	68	(17.3)
Good	102	92	194	(59.3)	88	118	206	(52.3)
Fair	36	35	71	(21.7)	36	57	93	(23.6)
Poor	8	4	12	(3.7)	9	17	26	(6.6)

**Table 3:** The number and percentage of respondents who answered more than once a week to the following question: “In the last six months, how often have you had the following …?”.

Physical Condition	Boy				*p*-Value	Girl				*p*-Value
	Niigata		Khabarovsk			Niigata		Khabarovsk		
	*n* = 176		*n* = 169			*n* = 151		*n* = 225		
Headache	29	(8.4)	47	(13.6)	0.103	36	(9.6)	47	(12.5)	0.477
Stomach-ache	16	(4.6)	25	(7.2)	0.644	15	(4.0)	37	(9.8)	0.073
Backache	23	(6.7)	32	(9.4)	0.118	18	(4.8)	62	(16.5)	<0.001
Feeling low	41	(12.1)	50	(14.8)	0.117	48	(12.8)	103	(27.4)	0.007
Irritability or bad temper	43	(12.6)	49	(14.3)	0.289	42	(11.2)	84	(22.3)	0.055
Feeling nervous	22	(6.5)	44	(12.9)	<0.001	22	(5.9)	97	(25.8)	<0.001
Difficulties in getting to sleep	17	(5.0)	32	(9.5)	0.009	8	(2.1)	76	(22.3)	<0.001
Feeling dizzy	25	(7.4)	33	(9.7)	0.197	21	(5.6)	37	(9.8)	0.504

**Table 4:** “Have you adapted to a new lifestyle to prevent the spread of infection?”.

	Boy				*p*-Value	Girl				*p*-Value
	Niigata Prefecture		Khabarovsk Region			Niigata Prefecture		Khabarovsk Region		
	*n* = 174		*n* = 169			*n* = 150		*n* = 224		
There was no problem because I didn’t feel much change	57	(32.8)	66	(39.1)	<0.001	23	(15.3)	79	(35.3)	<0.001
There was no problem because I was able to adapt comfortably	75	(43.1)	52	(30.8)		60	(40.0)	83	(37.1)	
It was difficult to adapt, but I was able to adapt	33	(19.0)	36	(21.3)		59	(39.3)	48	(21.4)	
It was difficult because I couldn’t stand the adaptation	9	(5.2)	15	(8.9)		8	(5.3)	14	(5.2)	

**Table 5:** “Are you afraid to get infected with COVID-19?”.

	Boy				*p*-Value	Girl				*p*-Value
	Niigata Prefecture		Khabarovsk Region			Niigata Prefecture		Khabarovsk Region		
	*n* = 176		*n* = 169			*n* = 151		*n* = 225		
I think it’s just a cold, so I’m not too scared	11	(6.3)	88	(52.1)		6	(4.0)	91	(40.4)	
I’m scared, but I’m ready to get infected	47	(26.7)	55	(32.5)	<0.001	26	(17.2)	96	(42.7)	<0.001
Very scared	113	(64.2)	24	(14.2)		119	(78.8)	36	(16.0)	

χ^2^-test; The differences between Niigata and Khabarovsk were compared by gender.

**Table 6:** Binormal regression of the responses to the question “Has the pandemic changed your self-assessment of health status?” (Niigata boys).

Factor	Partial Regression Coefficient	SD	Odds Ratio	95%CI
Intercept	−2.695 ***	0.470	0.068	[−3.708, −1.848]
Did not eat properly	1.100 *	0.462	3.003	[0.234, 2.065]
Difficult to adapt to a new lifestyle	1.070 *	0.432	2.915	[0.218, 1.923]
Don’t want to be vaccinated	0.694	0.479	2.001	[−0.270, 1.622]
Ready to get infected	0.709	0.404	2.032	[−0.594, 0.166]

*, *p* < 0.05, ***, *p* < 0.001. SD: standard deviation. CI: Confidence interval.

**Table 7:** Binormal regression of the responses to the question “Has the pandemic changed your self-assessment of health status?” (Khabarovsk boys).

Factor	Partial Regression Coefficient	SD	Odds Ratio	95%CI
Intercept	0.446	0.758	1.562	[−1.032, 1.960]
Did not eat properly	0.816	0.497	0.442	[−1.798, 0.169]
Lived a rhythmic life	−0.398	0.520	0.672	[−1.468, 0.592]
Exercised	−0.468	0.497	0.627	[−1.484, 0.484]
Did something to keep health	−0.639	0.462	1.895	[−0.234, 1.595]
Did not have any bad habits	−0.548	0.465	0.578	[−1.489, 0.346]
Difficult to adapt to a new lifestyle	1.193 **	0.396	3.300	[0.423, 1.982]
Don’t want to be vaccinated	−0.299	0.392	0.741	[−1.068, 0.476]
Ready to get infected	−1.169 *	0.490	0.311	[−2.144, −0.208]

*, *p* < 0.05, **, *p* < 0.01. SD: standard deviation. CI: Confidence interval.

**Table 8:** Binormal regression of the responses to the question “Has the pandemic changed your self-assessment of health status?” (Niigata girls).

Factor	Partial Regression Coefficient	SD	Odds Ratio	95%CI
Intercept	−1.501 ***	0.42	0.222	[−2.390, −0.730]
Did something to keep health	−0.650	0.445	1.915	[−0.194, 1.565]
Did not have any bad habits	−1.693	1.067	0.184	[−4.692, 0.008]
Don’t want to be vaccinated	0.661	0.447	1.936	[−0.226, 1.539]

***, *p* < 0.001. SD: standard deviation. CI: Confidence interval.

**Table 9:** Binormal regression of the responses to the question “Has the pandemic changed your self-assessment of health status?” (Khabarovsk girls).

Factor	Partial Regression Coefficient	SD	Odds Ratio	95%CI
Intercept	−1.232 ***	0.556	0.292	[−2.372, −0.150]
Did not eat properly	0.128	0.353	1.136	[−0.555, 0.836]
Lived a rhythmic life	0.3001	0.334	1.351	[−0.361, 0.954]
Exercised	−0.097	0.325	0.911	[−0.742, 0.535]
Did something to keep health	−0.688	0.339	1.894	[−0.011, 1.327]
Did not have any bad habits	−0.155	0.316	0.856	[−0.781, 0.462]
Difficult to adapt to a new lifestyle	−0.206	0.341	0.814	[−0.894, 0.450]
Don’t want to be vaccinated	0.912	0.319	0.901	[−0.726, 0.529]
Ready to get infected	0.912	0.410	1.046	[−0.737, 0.879]

***, *p* < 0.001. SD: standard deviation. CI: Confidence interval.

**Table 10:** Numbers and percentages of adolescents by nation and gender in response to the question “What do you think about the COVID-19 vaccination?”.

Gender	Prefecture or Region	Opt-In ^(1)^		Undecided ^(2)^		Opt-Out ^(3)^		*p*-Value
Boy	Niigata	132	(75.9) ^(4)^	9	(5.2)	33	(19.0)	<0.001
	Khabarovsk	63	(37.3)	63	(37.3)	43	(25.4)	
Girl	Niigata	117	(78.0)	26	(17.3)	7	(4.7)	<0.001
	Khabarovsk	77	(34.2)	87	(38.7)	61	(27.1)	
Total	Niigata	249	(76.9)	35	(10.8)	40	(12.3)	<0.001
	Khabarovsk	140	(35.5)	150	(38.1)	104	(26.4)	

^(1)^. Opt-in: Positively accept it or get vaccinated and will vaccinate when asked to vaccinate. ^(2)^. Undecided; Do not know and not bothered. ^(3)^. Opt-out; Unwilling and antivaccination. ^(^^4)^. The numbers in parentheses indicate percentages.

## Data Availability

The data that support the findings of this study are available from the corresponding author.
